# A follow-up study for left ventricular mass on chromosome 12p11 identifies potential candidate genes

**DOI:** 10.1186/1471-2350-12-100

**Published:** 2011-07-26

**Authors:** David Della-Morte, Ashley Beecham, Tatjana Rundek, Liyong Wang, Mark S McClendon, Susan Slifer, Susan H Blanton, Marco R Di Tullio, Ralph L Sacco

**Affiliations:** 1 Department of Neurology, Evelyn F. McKnight Brain Institute, Miller School of Medicine, University of Miami, Miami, FL, USA; 2 Department of Human Genetics, Hussman Institute for Human Genomics, Miller School of Medicine, University of Miami, Miami, FL, USA; 3 Department of Public Health and Epidemiology, Miller School of Medicine, University of Miami, Miami, FL, USA; 4 Department of Medicine, Columbia University, New York, NY, USA; 5 Department of Laboratory Medicine & Advanced Biotechnologies, IRCCS San Raffaele Pisana, Rome, Italy

## Abstract

**Background:**

Left ventricular mass (LVM) is an important risk factor for cardiovascular disease. Previously we found evidence for linkage to chromosome 12p11 in Dominican families, with a significant increase in a subset of families with high average waist circumference (WC). In the present study, we use association analysis to further study the genetic effect on LVM.

**Methods:**

Association analysis with LVM was done in the one LOD critical region of the linkage peak in an independent sample of 897 Caribbean Hispanics. Genotype data were available on 7085 SNPs from 23 to 53 MB on chromosome 12p11. Adjustment was made for vascular risk factors and population substructure using an additive genetic model. Subset analysis by WC was performed to test for a difference in genetic effects between the high and low WC subsets.

**Results:**

In the overall analysis, the most significant association was found to rs10743465, downstream of the *SOX5 *gene (p = 1.27E-05). Also, 19 additional SNPs had nominal p < 0.001. In the subset analysis, the most significant difference in genetic effect between those with high and low WC occurred with rs1157480 (p = 1.37E-04 for the difference in β coefficients), located upstream of *TMTC1*. Twelve additional SNPs in or near 6 genes had p < 0.001.

**Conclusions:**

The current study supports previously identified evidence by linkage for a genetic effect on LVM on chromosome 12p11 using association analysis in population-based Caribbean Hispanic cohort. *SOX5 *may play an important role in the regulation of LVM. An interaction of *TMTC1 *with abdominal obesity may contribute to phenotypic variation of LVM.

## Background

Increase in left ventricle mass (LVM) is considered a compensatory process which maintains cardiac function in response to noxious stimuli, such as hypertension, obesity and heart damage [1]. Increase in LVM is one of the most important cardiac risk factors for stroke and cardiovascular disease (CVD), including myocardial infarction (MI), and chronic heart failure (HF), independent of age, sex and race-ethnicity [2]. Previously in the Northern Manhattan Study (NOMAS), we demonstrated that LVM was significantly associated with risk of stroke, especially in Caribbean Hispanic individuals [3].

Hypertension, obesity, and diabetes are the most important determinants for hypertrophy of the left ventricle (LVH) [4]. Many genes related to these and other vascular risk factors may act independently or synergistically to increase risk for LVH. However, many subjects develop LVH and subsequently heart dysfunction by a process that remains poorly understood [1]. There is substantial variability in the risk to develop LVH at equal blood pressure or other risk factor levels, suggesting that LVH is a complex disorder not simply related to known vascular risk factors [5]. For this reason, a significant effort has been made to identify the genetic components underlying LVH. Studies performed in twins clearly established the heritability of LVM [6], which ranges from 0.3 to 0.7 in different study populations [7]. In Caribbean Hispanics from the Northern Manhattan Family Study, the LVM heritability ranged from 0.23 to 0.49 for the different left ventricle phenotypes [8]. The heritability for LVM was 0.58 among Dominican families [9].

Recently, we mapped a novel quantitative trail locus (QTL) for LVM to chromosome (Ch) 12p11 (MLOD = 3.11, p = 0.0003, peak marker = DS12S1042) independently of traditional cardiovascular risk factors among 1360 individuals from 100 Dominican families in the Family Study of Stroke Risk and Carotid Atherosclerosis [10]. The evidence for linkage was significantly increased (MLOD = 4.45, p = 0.0045 for increase in evidence of linkage) in a subset of families with high average waist circumference (WC) [9]. In the current study we present association analyses of single nucleotide polymorphisms (SNPs) in the 1 LOD critical region under this previously reported linkage peak (Ch12p11) with LVM [9] in an independent NOMAS subset.

## Methods

### Subjects and Data collection

In NOMAS [11], a prospective cohort study, 1137 individuals with brain MRI data were genotyped in a genome-wide association study (GWAS) to primarily study subclinical brain phenotypes. This group provided a convenience sample to further investigate genetic association with LVM on Ch12. While the 100 probands in the Family Study were drawn from NOMAS as described previously [10], only 67 were included in the NOMAS subset of 1137. These 67 individuals were excluded in the current association analysis so as to create a subset which was independent of the Family Study of Stroke Risk and Carotid Atherosclerosis. Also, because these NOMAS samples were genotyped primarily to study subclinical brain phenotypes, some samples did not have LVM data collected (N = 86) and were therefore dropped from analysis in addition to samples dropped because of genotyping quality control as described below (N = 87). Therefore, a total of 897 unrelated NOMAS individuals who were independent of the Family Study were available for the final analysis. The study was approved by the Institutional Review Boards of Columbia University, University of Miami, and the Independent Ethics Committee of Instituto Oncologico Regional del Cibao in the Dominican Republic. All subjects provided informed consent to participate.

### Phenotyping

Baseline transthoracic echocardiography was done on all 897 individuals in our NOMAS subset. Standard two-dimensional echocardiography, including colour flow and spectral Doppler examination, was performed according to the guidelines of the American Society of Echocardiography [12]. Special attention was paid to obtaining high quality parasternal long axis views of the left ventricle, from which left ventricular end-diastolic diameter (LVDD), left ventricular end-systolic diameter (LVSD), interventricular septum (IVS), and posterior wall thickness (PWT) were derived [13]. Sonographer performance was monitored quarterly after review of a random sample for technical adequacy of the images. Readers were blinded to vascular risk factors. Inter-observer variability for the variables of interest ranged between 8% and 10%. LVM was calculated according to the modified American Society of Echocardiography formula: [14].

### Genotyping and Quality Control

Genotyping was performed using the Genome-Wide Human SNP Array 6.0 chip (AffyMetrix). Samples were excluded because of failed genotyping in the lab or call rates below 95% (N = 44), relatedness because of unintentional enrollment of a parent, sibling, aunt/uncle (N = 22), gender discrepancies (N = 16), or were outliers beyond 6 SD from the mean based on Eigenstrat analysis (N = 5) [15]. SNPs with severe deviation from Hardy-Weinberg equilibrium (p < 10^-6^) or a genotyping call rate less than 95% were removed using PLINK 1.05 [16].

### Statistics

In the 1 LOD down critical region as identified in the family study on Ch 12p11 (MLOD = 3.11) [9], linear regression was performed on the 897 NOMAS individuals with PLINK using an additive genetic model. After quality control, genotype data were available on 7085 SNPs in the region from 23 to 53 MB, which includes 2 MB on either side of the 1 LOD down critical region. We did not limit this analysis to the 1 LOD critical region of the peak from a subset of families with high WC (MLOD = 4.45) based on the assumption that there may be multiple loci associated with LVM under our linkage peak, some of which contribute to an increase in LVM regardless of WC and others which contribute to an increase in LVM only in the presence of high WC. To reduce potential bias due to population stratification, we first performed principal component analysis to examine population substructure using EIGENSTRAT and selected the top two principal components (PCAs) as covariates for genomic control. Additionally, a covariate screening was done on risk factors such as age, sex, smoking, diabetes, dyslipidemia, hypertension, WC, and body mass index (BMI) using a stepwise selection procedure, and any with p < 0.10 were included as covariates in the final model. The number of years between risk factor information collection and LVM measurement was also included as a covariate. LVM measurements were natural log transformed to be consistent with our previous family analysis. Smoking was defined as never versus ever. Dyslipidemia was defined as a history of hyperlipidemia or total cholesterol greater than 240 mg/dL. Diabetes was defined as a history of diabetes or fasting blood sugar greater than 126 mg/dL, or use of insulin or hypoglycemic medications. Hypertension was defined as systolic blood pressure (SBP) ≥ 140, diastolic blood pressure (DBP) ≥ 90, history of hypertension, or on hypertensive medications. One of these conditions was sufficient to establish the diagnosis of hypertension.

In order to follow-up on the ordered subset analysis (OSA) used in the family study, which demonstrated a significant increase in the LOD score on Ch 12p11 in a subset of families with high WC [9], a subset analysis was also performed in the NOMAS subset of 897. High WC was defined as ≥ 40 inches in men and ≥ 35 inches in women according to the Third Report of the National Cholesterol Education Program - Adult Treatment Panel III (NCEP-ATP-III) [17]. Linear regression analysis was performed with PLINK, separately in the high (N = 495) and low WC (N = 402) subsets. An additive genetic model was used with the same covariate adjustments. After quality control, genotype data were available on 4334 SNPs in the region from 23 to 41 MB, which includes 2 MB on either side of the 1 LOD down critical region of the OSA peak. To test the difference in effect size between the high and low WC subsets, or a WC*SNP interaction in association with LVM, a Z-score was computed for the difference in β coefficients and significance assessed.

To correct for multiple testing of SNPs, we applied SimpleM [18]. SimpleM estimates the number of independent tests such that a standard Bonferroni correction can be applied while maintaining the prescribed level of α. The effective number of independent tests is 4730 for the 7085 SNPs surveyed. Using standard Bonferroni correction, the peak-wide significance threshold is 1.06E-05. For the 1 LOD critical region of the OSA peak, the effective number of independent tests is 2883 for the 4334 SNPs surveyed. Using standard Bonferroni correction, the peak-wide significance threshold is 1.73E-05.

Quanto [19] was used to calculate statistical power. Assumptions included independence of individuals, MAF of 0.20, an additive genetic effect, a population mean of 5.22 and standard deviation of 0.27 for the natural logarithm of LVM (estimated in the 897 NOMAS subjects used in the final analysis), and a two-sided α of 0.001. With 897 multi-ethnic samples from NOMAS we had over 80% power to detect an effect size of 0.065 (corresponding to an approximate change of 12 grams from the mean of LVM). Given the above assumptions, in addition to assuming a population prevalence of 0.55 for high waist circumference, we had over 80% power to detect a difference in beta coefficients of 0.125 between the high and low WC subsets.

## Results

The NOMAS cohort is mainly composed of Caribbean Hispanics (65%), with most Hispanics being Dominican (64%). Table 1 summarizes the sociodemographic, vascular risk factors, and LVM in the final NOMAS sample.

**Table 1:** Sociodemographics, vascular risk factors, and LVM measurements among 897 subjects from NOMAS

	n	%
Hypertension	569	63.4
Diabetes	178	19.8
Ever Smoking	469	52.3
Dyslipidemia	427	47.9
Race		
White	140	15.6
Black	156	17.4
Hispanic	583	65.0
Other	18	2.0
Sex		
Male	368	41.0
Female	529	59.0
		

	**Mean ± SD**	

Age	70.8 ± 8.9	
Body mass index (kg/m2)	28.3 ± 4.9	
Waist circumference (inch)	37.8 ± 4.8	
Fast glucose (mg/dl)	101.2 ± 33.6	
Total cholesterol (mg/dl)	194.2 ± 39.7	
LDL (mg/dl)	115.3 ± 35.6	
HDL (mg/dl)	53.8 ± 17.4	
Triglyceride (mg/dl)	127.0 ± 81.9	
SBP (mmHg)	136.4 ± 17.4	
DBP (mmHg)	78.0 ± 9.6	
Pack years among smokers	22.9 ± 26.4	
Years b/w left ventricular mass and covariate measurements	0.6 ± 2.1	
Left ventricular mass (g)	191.8 ± 53.9	

**Figure 1:**
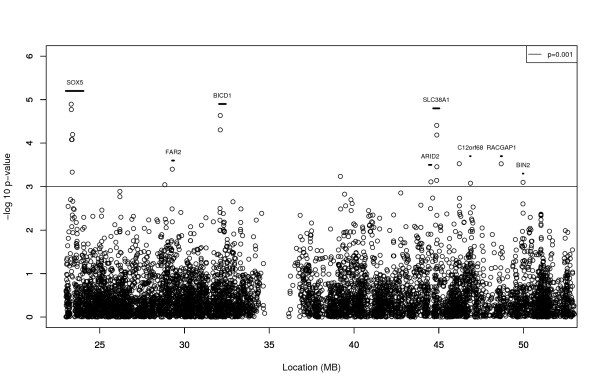
**Peak wide association results on Chromosome 12p11**. The region shown (23-53 MB) is approximately the 1 LOD down region of the multipoint linkage analysis in the Family Study of Stroke Risk and Carotid Atherosclerosis.

For association analysis, a total of 7085 SNPs were located within the 23 to 53 MB critical region. Covariates included age, sex, WC, BMI, hypertension, and diabetes, in addition to years between risk factor and LVM measurement, PCA1, and PCA2. The top associated SNP, rs10743465 (p = 1.27E-05, beta=-0.066) is located downstream of sex determining region Y-box 5 (*SOX5*). There are nineteen SNPs with a nominal p ≤ 0.001, more than the seven expected by chance alone, but not meeting peak-wide significance which reside in potential candidate genes for LVM; including Solute carrier family 38, member 1 (*SLC38A1*) and Bicaudal D homolog 1 (*BICD1*) (Table 2 and Figure 1).

**Table 2:** Top associated SNPs (p < 00.001) in the overall sample on chromosome 12p11

					Overall Data Set (N = 897)				Dominican Subset (N = 368)			
**SNP**	**MB**	**Gene**	**Function**	**Minor Allele**	**MAF**	**Beta**	**SE**	P*	**MAF**	**Beta**	**SE**	**P†**

rs10743465	23.315	SOX5	flanking	A	0.202	-0.066	0.015	1.27E-05	0.23	-0.043	0.022	5.11E-02
rs4321001	23.323	SOX5	flanking	A	0.195	-0.064	0.015	1.69E-05	0.22	-0.036	0.021	9.76E-02
rs16919217	32.110	BICD1	flanking	C	0.079	-0.085	0.020	2.31E-05	0.11	-0.082	0.027	2.68E-03
rs6582621	44.878	SLC38A1	intron	A	0.268	-0.054	0.013	3.91E-05	0.29	-0.062	0.020	2.17E-03
rs16919218	32.110	BICD1	flanking	T	0.082	-0.081	0.020	4.96E-05	0.11	-0.082	0.027	2.99E-03
rs7958592	23.399	SOX5	flanking	C	0.340	-0.048	0.012	6.33E-05	0.38	-0.047	0.018	7.80E-03
rs11183394	44.882	SLC38A1	intron	A	0.239	-0.054	0.013	6.50E-05	0.25	-0.060	0.021	4.30E-03
rs10047623	23.368	SOX5	flanking	G	0.092	-0.078	0.020	8.28E-05	0.12	-0.066	0.027	1.70E-02
rs4129991	23.340	SOX5	flanking	G	0.202	-0.059	0.015	8.28E-05	0.23	-0.038	0.021	7.54E-02
rs7307902	46.209	intergenic		G	0.213	0.050	0.014	2.98E-04	0.19	0.022	0.022	3.14E-01
rs1967110	48.681	RACGAP1	intron	G	0.086	-0.075	0.021	2.99E-04	0.09	-0.063	0.031	3.86E-02
rs7133522	44.888	SLC38A1	intron	T	0.292	-0.046	0.013	3.48E-04	0.31	-0.055	0.019	4.53E-03
rs12310555	29.278	FAR2	intron	A	0.189	-0.053	0.015	3.97E-04	0.21	-0.083	0.023	2.88E-04
rs7955257	23.376	SOX5	flanking	T	0.260	-0.044	0.013	4.64E-04	0.28	-0.038	0.019	4.78E-02
rs17128396	39.198	intergenic		A	0.063	-0.082	0.024	5.80E-04	0.07	-0.055	0.034	1.03E-01
rs7956629	44.877	SLC38A1	intron	A	0.120	-0.061	0.018	7.18E-04	0.14	-0.065	0.026	1.27E-02
rs7310702	44.535	ARID2	intron	G	0.168	-0.055	0.016	7.78E-04	0.19	-0.044	0.023	5.90E-02
rs12227330	49.969	BIN2	intron	A	0.121	-0.063	0.019	7.95E-04	0.12	-0.062	0.028	2.98E-02
rs10875764	46.869	C12orf68	flanking	C	0.482	0.039	0.012	8.34E-04	0.46	0.035	0.019	5.94E-02
rs309045	28.841	intergenic		A	0.413	0.039	0.012	9.07E-04	0.36	0.017	0.018	3.39E-01

*adjused for PCA1, PCA2, age, sex, hypertension, diabetes, BMI, WC, and years between LVM and risk factor measurements

† adjusted for PCA1, age, sex, hypertension, diabetes, BMI, WC, and years between LVM and risk factor measurements.

The NOMAS multi-ethnic sample of 897 individuals was used to maximize statistical power. However, to evaluate potential substructure bias, we have performed the same analyses in the Dominican subset only (368 individuals). The trends of association are all in the same direction for SNPs with p ≤ 0.001, as indicated by the β values (Table 2). While all but three of the twenty SNPs are still significant with p < 0.10 in the Dominican subset, the p-values are less significant. This is expected as we only have 26% power to detect an effect size of 0.065 in the Dominican subset, compared to over 80% power to detect the same effect size in the multi-ethnic NOMAS sample.

**Figure 2:**
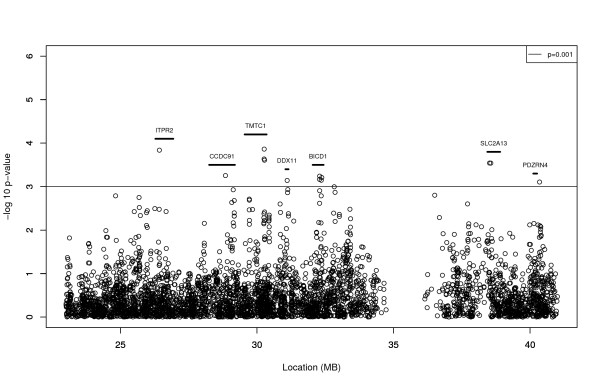
**Differential association with LVM among individuals with Low and High WC**. The region shown (23-41 MB) is approximately the 1 LOD down region of the high waist circumference OSA analysis in the Family Study of Stroke Risk and Carotid Atherosclerosis.

There were 495 individuals with high and 402 individuals with low WC. For the WC subset analysis, 4334 SNPs were located within the 23 to 41 MB critical region of the OSA peak. While no SNPs met the peak wide significance criterion of 1.73E-05 for the test of the difference in effect sizes between the high and low WC subset, there are thirteen SNPs with p ≤ 0.001, which is more than the five expected by chance alone (Table 3 and Figure 2). Also, most of the differences seen in beta coefficients between the high and low WC subsets were less than 0.125, and so we may not have had adequate power to detect the effects more significantly. Note that none of these SNPs are significant (p ≤ 0.001) in the overall sample (N = 895), and so their effect on LVM is masked without consideration of their interaction with WC. The most significant difference in genetic effect between individuals with high and low WC occurred with rs1124636 (p = 1.45E-04), located upstream of transmembrane and tetratricopeptide repeat containing 1 (*TMTC1*). Associations were seen in several other interesting candidate genes such as Inositol 1,4,5-triphosphate receptor type 2 (*ITPR2*) for SNP rs1124636 (p = 1.45E-04) and in multiple intronic SNPs of Solute Carrier Family 2 (facilitated glucose transporter), Member 13 (*SLC2A13) *and *BICD1 *(Table 3 and Figure 2).

**Table 3:** SNPs with differential association (p < 0.001) with LVM among High and Low Waist Circumference (WC)"

					Overall (N = 897)			High WC (N = 495)			Low WC (N = 402)				
**SNP**	**MB**	**Gene**	**Function**	**Minor Allele**	**Beta**	**SE**	P*	**Beta**	**SE**	P*	**Beta**	**SE**	P*	**Difference in Betas**	**P†**

rs1157480	30.264	TMTC1	flanking	A	0.004	0.014	7.88E-01	0.053	0.019	6.30E-03	-0.056	0.021	8.20E-03	0.109	1.37E-04
rs1124636	26.424	ITPR2	intron	T	-0.016	0.014	2.54E-01	0.032	0.019	9.31E-02	-0.072	0.020	2.85E-04	0.104	1.45E-04
rs9669515	30.261	TMTC1	flanking	T	0.002	0.014	8.74E-01	0.049	0.019	1.04E-02	-0.055	0.021	8.68E-03	0.104	2.31E-04
rs7970841	30.274	TMTC1	flanking	T	-0.007	0.015	6.31E-01	0.042	0.020	3.04E-02	-0.064	0.021	3.03E-03	0.106	2.46E-04
rs7963790	38.515	SLC2A13	intron	C	0.022	0.013	1.03E-01	0.064	0.017	2.40E-04	-0.030	0.019	1.20E-01	0.095	2.87E-04
rs10877703	38.561	SLC2A13	intron	A	0.012	0.013	3.37E-01	0.052	0.017	2.47E-03	-0.041	0.019	3.22E-02	0.093	2.87E-04
rs1679695	28.845	CCDC91	flanking	T	-0.044	0.022	5.20E-02	-0.106	0.028	2.12E-04	0.050	0.035	1.55E-01	0.156	5.56E-04
rs326644	32.293	BICD1	intron	C	-0.009	0.012	4.41E-01	0.024	0.016	1.34E-01	-0.059	0.018	1.23E-03	0.083	5.73E-04
rs261889	32.371	BICD1	intron	T	0.014	0.014	3.40E-01	-0.030	0.019	1.11E-01	0.066	0.021	1.65E-03	0.096	6.18E-04
rs326641	32.291	BICD1	intron	T	0.015	0.014	2.75E-01	0.059	0.018	1.23E-03	-0.032	0.020	1.06E-01	0.091	6.82E-04
rs2650128	32.352	BICD1	intron	G	0.014	0.015	3.32E-01	-0.026	0.019	1.77E-01	0.074	0.023	1.10E-03	0.100	7.10E-04
rs4356315	31.105	DDX11	flanking	G	0.014	0.012	2.38E-01	0.050	0.015	1.18E-03	-0.028	0.017	1.07E-01	0.078	7.21E-04
rs11610493	40.345	PDZRN4	flanking	G	0.014	0.013	2.57E-01	-0.023	0.017	1.70E-01	0.061	0.018	1.05E-03	0.084	7.82E-04

*adjusted for PCA1, PCA2, age, sex, hypertension, diabetes, BMI, WC, and years between LVM and risk factor measurements

† p-value calculated using a normal approximation to test for differences between the beta coefficients of high and low WC.

## Discussion

Using our well-characterized extended Dominican Republic families, we have previously mapped a novel QTL near D12S1042 on Ch 12p11 for LVM, with an increase in the evidence for linkage seen for a subset of families with high WC. This provides a well defined chromosome region and phenotype for validation [9]. In this study, we follow up with SNPs in the 1 LOD critical region of Ch 12p11 peak in an independent NOMAS subset. We found SNPs in or near several notable genes *(SOX5, SLC38A1, BICD1) *associated with LVM. A significant difference in genetic effects between individuals with high and low WC was seen for SNPs in or near *TMTC1, SLC2A13, ITPR2*, and *BICD1*.

LVH has been largely recognized as one of the most important independent risk factors for CVD and stroke [2]. LVH is associated with biochemical and molecular changes in myocardial cells. Changes in gene expression patterns play a pivotal role in determining the hypertrophic phenotype. A linkage study performed in a cohort of African-American hypertensive siblings from the Genetic Epidemiology Nertwork of Arteriopathy (GENOA) study demonstrated genetic linkage for LV structure in Ch 3, 12, and 19. Interestingly on Ch 12, the solute carrier family 15, member 4 (*SLC15A4*) was discussed as a possible candidate gene implicated in molecular mechanisms controlling the LV wall thickness, a precursor of LVM [20]. This gene has a function similar to *SLC38A1 *which was associated with LVM in the present study. Several other polymorphisms have been associated with LVM including polymorphisms in Transforming Growth Factor (TGF)-beta3 [21]; in insulin-like growth factor (IGF)-1 receptor gene [22]; in the G protein beta subunit (GNB3) gene [23]; and in the aldosterone synthase gene [24]. However, findings from different studies are often controversial and provide little or no overlap. For this reason, we performed validation of our LVM linkage in an association study of the SNPs in the region under the linkage peak in an independent sample derived from a multi-ethnic population.

While no markers met our peak-wide significance threshold, we found the most significant association to rs10743465, downstream of *SOX5*, an interesting candidate gene. This gene encodes transcription factors with a high-mobility-group (HMG) box DNA-binding domain similar to that of the sex-determining region (Sry) protein. *SOX5 *gene expression is modulated by Nitric Oxide (NO) and Guanylyl Cyclase after shear stress in endothelial cells [25]. Endothelial NO levels were significantly correlated with LVM [26]. In addition, *SOX5 *plays a pivotal role in the expression of the muscle L-type Ca^2+ ^channel or dihydropyridine-sensitive receptor (DHPR) [27]. Variation in the expression of these channels is associated with cardiac hypertrophy [28]. Recently, a meta-analysis of GWAS for the PR interval, a direct measurement of atrial and atrioventricular nodal conduction, implicated *SOX5 *to be most prominent gene in controlling the PR interval [29].

We found associations of LVM with SNPs located in or near *SLC38A1 *and *BICD1. E*xpression of *SLC38A1 *has been proposed as a marker for cardiac development [30]. *BICD1 *plays a role in controlling telomere length variation in humans, which is pivotal in controlling DNA replication and cellular proliferation [31]. Telomere length has been proposed as a new marker of CVD especially for its role in atherosclerotic process, arterial hypertension, and myocardial infarction [32]. Recently, BICD1 has been shown to directly modulate G protein signaling, cell proliferation, and endocytosis downstream of Protease-activated receptor-1 (PAR1) [33] that is involved in cardiomyocytes contractility dysfunction [34].

Association with LVM was also found in other notable genes such as Rac GTPase activating protein 1 (*RACGAP1)*, Open reading frame 68 (*C12orf68)*, and fatty acyl CoA reductase 2 (*FAR2*). The RACGAP1, has been implicated in mechanisms regulating cellular proliferation [35]. Similarly to SOX5, Ch12 C12orf68 also regulates the PR interval [29]. FAR2 plays an important role in the biosynthesis of functional lipids, such as phospholipids, through peroxisomal beta-oxidation [36]. The cardiomyocytes body grows when it is present a deregulation in the synthesis of phospholipids [37].

Complex molecular mechanisms lead to LVH, such as fibrotic changes in the extracellular matrix, adaptive cellular changes within the sarcomere, biochemical and molecular changes in myocardial cells [7]. In addition, hemodynamic mechanisms have a direct functional effect on LVH [26]. Therefore, genes that encode for proteins regulating LV structure, calcium homeostasis, substrate metabolism, growth factors, cell signaling, and hemodynamic mechanisms are promising candidate genes.

Previously we reported a significant increase of the LOD score on Ch 12p11 in a subset of families with high WC [9]. The relationship between LVM and WC or visceral obesity has been demonstrated, especially in women [38,39]. Moreover, a study conducted in a population of 341 twins demonstrated the genetic correlation between weight and LVM. The mechanism seems related to an increase in sympathetic activity and insulin resistance [40].

In the present study, the most significant difference in genetic effect between individuals with high and low WC occurred in SNPs flanking *TMTC1*. In a meta-analysis of GWAS, rs2046383 in *TMTC1 *has been associated with heart failure in an African ancestry population [41]. Moreover, *TMTC1 *genetic variants have been associated with modulation in lipids metabolism [42]. These findings may explain, at least in part, its relationship with LVM and WC.

Other differential associations with LVM between individuals with high and low WC were found in notable genes such as *SLC2A13, ITPR2*, and *BICD1. SLC2A13 *regulates glucose viability through the tissue, which might in part explain its role in the relationship between WC and LVM [43]. *ITPR2 *has been associated with greater SBP [44], which is a risk factor for both LVM and increased WC. The main role of the protein synthesized by *ITPR2 *is to regulate the Ca^2+ ^fluxes in myocytes [45]. The alteration in ITPR2 is coupled with initiation and/or progression of hypertrophy and heart failure [46]. An in vivo study showed difficulties in nutrient digestion and metabolism in *ITPR2 *knockout mice compared to wild type due to the lack of Ca^2+ ^signaling in exocrine tissues [47]. As previously mentioned, *BICD1 *plays a role in controlling telomere length variation in humans and for this activity might play a role in LVM. However, telomere length variation has been also associated with insulin resistance, oxidative stress, and uncoupling protein 2 (UCP2), all of which one related to fat metabolism [48].

There are several strengths of our study. First, the LVM assessment was performed by the same investigators in all patients, adopting a common protocol to assure consistent phenotyping. Second, we followed the genome-wide linkage approach by high-resolution association analysis in an independent cohort, which allowed for evaluation of the genetic contribution to LVM through the genome. We also acknowledge several limitations. There is the possibility that other covariates or confounders may not have been evaluated. However, we included the most well established risk factors for LVH. Finally, our validation sample was composed primarily of Hispanics. The average age of our population was around 71 years, and therefore we risk potential bias of studying survivors. As a result, our findings may not be directly generalized to other populations.

## Conclusion

LVH has been identified as an independent risk factor for stroke and cardiovascular disease. Previously we found evidence for linkage for LVM on Ch 12p11 [9]. In the present peak-wide association study, we identified suggestive evidence of novel SNPs located on Ch 12 associated with LVM. The candidate genes, such as *SOX5 *reported here, may have important functional relevance for LVM. In addition, an interaction of genes, e.g. *TMTC1 *with abdominal obesity, may contribute to phenotypic variation of LVM. While our results did not reach peak-wide significance, they point to interesting candidate genes. Confirmation studies are needed to verify these associations.

## List of Abbreviations

*ARID2: *AT Rich Interactive Domain 2; ARVD/C: Arrhythmogenic Right Ventricular Dysplasia/Cardiomyopathy; *BHLHE41: *Basic Helix-Loop-Helix Family, Member e41; *BICD1: *Bicaudal D Homolog 1; BMI: Body Mass Index; *BIN2: *bridging integrator 2; Ch: Chromosome; *CCDC91: *Coiled-Coil Domain Containing 91; *C12orf68: *Open Reading Frame 68*; *CVD: Cardiovascular Disease; *DDX11: *DEAD/H Box Polypeptide 11; DHPR: Dihydropyridine-Sensitive Receptor; *FAR2: *Fatty Acyl CoA Reductase 2; GENOA: Genetic Epidemiology Nertwork of Arteriopathy; GNB3: G Protein Beta Subunit; GWAS: Genome-Wide Association Study; HF: Heart Failure; HMG: High-Mobility-Group; IGF-1: Insulin-Like Growth Factor 1; *ITPR2: *Inositol 1,4,5-Triphosphate Receptor Type 2; IVS: Interventricular Septum; LOD: Logarithm of the Odds; LVDD: Ventricular End-Diastolic Diameter; LVH: Hypertrophy of the Left Ventricle; LVM: Left Ventricular Mass; LVSD: Left Ventricular End-Systolic Diameter; MI: Myocardial Infarction; NCEP-ATP-III: Third Report of the National Cholesterol Education Program - Adult Treatment Panel III; NO: Nitric Oxide; NOMAS: Northern Manhattan Study; OSA: Ordered Subset Analysis; PCAs: Principal Components; *PDZRN4: *PDZ Domain Containing Ring Finger 4; *PKP2: *Plakophilin 2; PWT: Posterior Wall Thickness; PAR1: Protease-Activated Receptor-1; QTL: Quantitative Trail Loci; *RACGAP1: *Rac GTPase Activating Protein 1; SBP: Systolic Blood Pressure; *SLC2A13: *Solute Carrier Family 2 (facilitated glucose transporter) Member 13; *SLC38A1: *Solute Carrier Family 38, Member 1; *SLC15A4: *Solute Carrier Family 15, Member 4; SNPs: Single Nucleotide Polymorphisms; *SOX5: *Sex Determining Region Y-box 5; Sry: Sex-Determining Region; WC: Waist Circumference; TGF: Transforming Growth Factor; TMTC1*: *Tetratricopeptide Repeat Containing 1; UCP2: Uncoupling Protein 2.

## Competing interests

The authors declare that they have no competing interests.

## Authors' contributions

All authors read and approved the final manuscript. DDM: contributed to interpretation the data, drafting the manuscript, critical review of the manuscript. AB: analyzed and interpreted the data, drafting the manuscript, critical review of the manuscript. TR: contributed to planning of the study, study design, interpretation of the data, writing the manuscript. LW: analyzed and interpreted the data, critical review of the manuscript. SS: analyzed and interpreted the data, drafting the manuscript. MSMC: analyzed and interpreted the data, drafting the manuscript. SHB: contributed to planning of the study, interpretation of the data, writing the manuscript. MRDT: contributed to planning of the study, interpretation of the data, writing the manuscript. RLS: contributed to planning of the study, analysis and interpretation the data, study conduct, writing and critical review of the manuscript.

## Acknowledgements and Funding

This work was supported by grants from the National Institute of Neurological Disorders and Stroke (R01 NS NS40807, R01 NS047655, R37 NS29993) and the Evelyn F. McKnight Brain Institute.

## Pre-publication history

The pre-publication history for this paper can be accessed here:

http://www.biomedcentral.com/1471-2350/12/100/prepub
